# *Campylobacter* spp. in Poultry Slaughterhouses: Occurrence, Antimicrobial Resistance, and Virulence-Associated Genes

**DOI:** 10.3390/pathogens15060585

**Published:** 2026-05-29

**Authors:** Pietro Di Taranto, Fiorenza Petruzzi, Antonella Cristina Romano, Mariateresa Toce, Lucia Palazzo, Alessandra Alessiani, Loredana Capozzi, Stefano Castellana, Laura Del Sambro, Adelia Donatiello, Carmine Pedarra, Gilda Occhiochiuso, Giovanni Castelli, Alessandra Barlaam, Giovanni Normanno, Antonio Parisi

**Affiliations:** 1 Istituto Zooprofilattico Sperimentale della Puglia e della Basilicata, Via Manfredonia 20, 71121 Foggia, Italy; pietro.ditaranto@izspb.it (P.D.T.); antonella.romano@izspb.it (A.C.R.); mariateresa.toce@izspb.it (M.T.); lucia.palazzo@izspb.it (L.P.); loredana.capozzi@izspb.it (L.C.); stefano.castellana@izspb.it (S.C.); adelia.donatiello@izspb.it (A.D.); carmine.pedarra@izspb.it (C.P.); gilda.occhiochiuso@izspb.it (G.O.); giovanni.castel@gmail.com (G.C.); antonio.parisi@izspb.it (A.P.); 2 Istituto Zooprofilattico Sperimentale dell’Abruzzo e del Molise “G. Caporale”, Via Campo Boario, 64100 Teramo, Italy; a.alessiani@izs.it; 3 Dipartimento di Scienze Agrarie, Alimenti, Risorse Naturali e Ingegneria, Università degli Studi di Foggia, Via Napoli 25, 71121 Foggia, Italy; alessandra.barlaam@unifg.it (A.B.); giovanni.normanno@unifg.it (G.N.)

**Keywords:** *Campylobacter jejuni*, *Campylobacter coli*, foodborne pathogens, food safety monitoring, antimicrobial resistance

## Abstract

Poultry is the main reservoir of *Campylobacter* spp. and most human cases result from consuming undercooked poultry or handling raw meat. In 2022, a total of 55 samples, including neck skin, cecal contents, and processing waters, were collected at two poultry slaughterhouses in Italy and analysed according to ISO 10272-2:2017 at the Istituto Zooprofilattico Sperimentale della Puglia e della Basilicata laboratories. Overall, 51/55 (92.72%) samples tested positive for *Campylobacter*. Among the isolates, 64.71% were identified as *C. coli*, and 35.29% as *C. jejuni*. Phenotypic and genotypic analysis were performed to assess antimicrobial resistance and virulence characteristics. All *C. jejuni* isolates and 72.72% of *C. coli* showed resistance to fluoroquinolones. Resistances to tetracycline and carbapenem were observed in 60.78% and 45.09% of isolates, respectively. Genomic analysis confirmed the presence of the *tet(O)* gene, conferring tetracycline resistance. In addition, *OXA-450* and *OXA-466* genes, conferring beta-lactam resistance, were detected in 78.43% and 3.92% of isolates. Virulence-associated genes were detected. Specifically, the *ciaB* gene was found in 50/51 (98.04%) of isolates, whereas *jlpA*, *cdtA*, *cdtB*, and *ctdC* genes were exclusively identified in *C. jejuni* strains. The high prevalence of pathogenic and antimicrobial-resistant *Campylobacter* strains highlights the need for strengthened control measures along the poultry production chain.

## 1. Introduction

*Campylobacter* spp. are Gram-negative curved rod-shaped bacteria, motile due to the presence of a polar flagellum [1]. The genus *Campylobacter* is classified into five phylogenetic groups comprising 57 species. Among them, several species can cause disease but *C. jejuni* and *C. coli* are most frequently implicated in human infections, with a clear prevalence of *C. jejuni* (80–85%) [2,3].

Campylobacteriosis is typically characterised by acute and self-limiting gastrointestinal disorders, such as diarrhoea, fever and abdominal pain [4]. However, more severe complication, including Guillain–Barrè syndrome or reactive arthritis may occur, particularly in young children, the elderly, and immunocompromised people, potentially leading to the death [5].

From a food safety perspective, *Campylobacter* spp. is the most commonly reported foodborne pathogen in Europe [6]. In 2021, campylobacteriosis was the most reported foodborne gastrointestinal infection in humans in Europe, with 249 notified outbreaks, 127,840 cases of illness, 10,469 hospitalizations, and 26 deaths [7].

Moreover, in the last five years the number of campylobacteriosis cases reported by the European Food Safety Authority (EFSA) and the European Centre for Disease Prevention and Control (ECDC) showed a significant increasing trend, followed by salmonellosis, listeriosis, and Shiga toxin-producing *Escherichia coli* (STEC) infections [8].

Infection is primarily transmitted through the consumption of contaminated food, including raw or undercooked meat, unpasteurised milk, vegetables, and water, or contact with infected animals or contaminated environments [2]. However, several studies reported that the most frequent causes of *Campylobacter* infection are poultry and poultry products. Poultry is considered as a natural reservoir, to which 50–80% of cases in the European Union (EU) may be attributed [9,10].

Although poultry is frequently colonised with *Campylobacter* spp. in the intestines without showing clinical symptoms, the contamination of the poultry carcasses may occur during slaughter and processing due to improper hygiene practices. This can enable cross-contamination and the spread of the bacteria in the poultry chain [11]. Moreover, poor consumer practices in handling raw meat and inadequate cooking are considered the main risk factors for the onset of campylobacteriosis, especially given the low infectious dose [12].

To control this source of contamination, the legislator added a new process hygiene criterion in EU food law, based on the enumeration of *Campylobacter* spp. in broiler neck skin samples collected during slaughter.

Specifically, Regulation (UE) 1495/2017, amending the Regulation (EC) n. 2073/2005, dictates that the hygiene of the slaughtering process is considered satisfactory if no more than 15 out of 50 samples (30%) exceed 1000 colony-forming units (CFU)/g of neck skin for *Campylobacter* [13,14].

Antimicrobial-resistance (AMR) in *Campylobacter* spp. is an emerging global concern [15]. Although most cases of human campylobacteriosis are treatable, an increasing concern has been raised about the ability of *Campylobacter* to develop resistance to several classes of antibiotics used in human and veterinary medicine, including fluoroquinolone, macrolides, and tetracycline [16].

From an epidemiological perspective, in order to investigate genetic correlations between isolates, whole genome sequencing (WGS) followed by multilocus genotyping (using core genome Multi Locus Sequence Typing, cgMLST) are essential tools. Resistome and virulome analysis conducted using WGS can also provide further insights into antimicrobial resistance and virulence characteristics [17,18].

Starting from these assumptions, this study aimed to investigate the occurrence of *Campylobacter* spp. in samples collected at two poultry slaughterhouses located in the Basilicata region (Southern Italy), and characterise the antimicrobial resistance and virulence profiles of the isolates.

## 2. Materials and Methods

### 2.1. Sample Collection

From June to November 2022, 55 samples were collected at two poultry slaughterhouses located in Picerno (PZ) (site A) and Abriola (PZ) (site B) (Basilicata region). Specifically, 10 neck skin samples and 16 cecum samples were collected from each slaughterhouse. Each neck skin sample consisted of neck skins collected at random from five poultry carcasses from the same flock after chilling. In addition, three water samples used to wash poultry’s carcasses were collected at the slaughterhouse located in site A.

The samples were sent to the laboratories of the Istituto Zooprofilattico Sperimentale della Puglia e della Basilicata (IZSPB) at a temperature ranging between 1 °C and 8 °C, and the time between sampling and analysis was less than 48 h, in order to ensure sample integrity. All neck skin samples were analysed according to ISO 10272-2:2017.

### 2.2. Bacterial Detection

The content from each cecum sample was taken with a 10 μL sterile loop and seeded directly onto modified charcoal cefoperazone deoxycholate agar plates (mCCD Agar; Biolife Italiana srl, Milan, Italy). The plates were incubated at 41.5 °C ± 1 °C for 44 ± 4 h in a microaerophilic condition. Water samples were analysed as cecum content. After the incubation period, the plates were examined for the presence of typical colonies. For each positive plate, up to five presumptive *Campylobacter* colonies with the typical morphology were subcultured in Columbia blood agar plates (Meus srl, Taranto, Italy) and incubated under microaerophilic conditions at 41.5 ± 0.5◦C for 48 h. Isolates were identified to the genus level using MALDI-TOF MS (Matrix-Assisted Laser Desorption/Ionisation—Time of Flight Mass Spectrometry), following the producer guidelines (Bruker Daltonics GmbH, Bremen, Germany) and then confirmed by biochemical tests (oxidase test), the study of aerobic growth at 25 °C for 44 ± 4 h, microscopic examination, and gram staining. *Campylobacter* spp. colonies were counted, and the results were expressed as colony-forming units per gram of sample (CFU/g). One isolate per positive sample was stored at −80 °C in Microbank™ vials (Pro-Lab Diagnostics, Richmond Hill, ON, Canada).

### 2.3. Antimicrobial Susceptibility Testing

One confirmed *Campylobacter* spp. isolate per positive sample was subjected to a phenotypic antimicrobial susceptibility test using the microbroth dilution method. It was performed with an antimicrobial panel (EUCAMP3^®^, Thermo Fisher Scientific, Paisley, UK) used in the official antimicrobial resistance monitoring scheme in the EU (Commission Implementing Decision (EU) 2020/1729) [19].

Briefly, the *Campylobacter* isolates were transferred to 10 mL of cation adjusted Mueller–Hinton (CAMHB; Becton Dickinson, Milan, Italy) broth that contained 5% of laked horse blood (Oxoid, Basingstoke, Hampshire, England) and incubated at 37 ± 1 °C under microaerophilic conditions for 20 ± 2 h. Then, 50 μL of bacterial suspension were transferred into each well of the working plates (EUCAMP3^®^, Thermo Fisher Scientific, Paisley, UK). The plates were incubated at 37 ± 1 ° C for 40–48 h in microaerophilic conditions before the results were recorded. *Staphylococcus aureus* ATCC^®^ 29213 (ATCC; Manassas, VA, USA) was used for quality control as previously described.

The EUCAMP3^®^ plates contained a panel of six antibiotics. More specifically, chloramphenicol (CHL; 2–64 µg/mL), erythromycin (ERY; 1–512 µg/mL), gentamicin (GEN; 0.25–16 µg/mL), ciprofloxacin (CIP; 0.12–32 µg/mL), tetracycline (TET; 0.5–64 µg/mL), and ertapenem (ETP; 0.12–4 µg/mL).

The lowest concentration that inhibited the growth of bacteria was considered as the minimum inhibitory concentration (MIC).

The plates were read in a Sensititre™ Vizion System (Thermo Fisher Scientific, Paisley, UK).

Breakpoints used for the interpretation of the results were based on the epidemiological cut-off value (ECOFF), as shown in Report n. 13 Breakpoints EUCAST (European Committee on Antimicrobial Susceptibility Testing), in order to classify the microorganism as susceptible (S) or resistant (R) to each antibiotic [20].

Isolates that demonstrated resistance to three or more antimicrobials were categorised as multidrug-resistant (MDR).

### 2.4. Genome Sequencing

All isolated strains identified by microbiological methods were subjected to molecular analysis.

Genomic DNA was extracted from each strain using the DNeasy Blood & Tissue Kit (Qiagen, Hilden, Germany), according to the manufacturer’s instructions.

Starting from the purified genomic DNA, an indexed genomic library for each sample was prepared using the Illumina DNA Prep Sample Preparation Kit (Illumina, San Diego, CA, USA), as previously described [21]. A 2 × 250 paired-end sequencing run was performed on the Illumina MiSeq platform.

Raw sequences obtained were checked by FastQC v0.12 application, while read filtering/trimming and *de novo* assemblies were performed in the Galaxy environment using fastp v1.0.1 and SPAdes v3.15 tools [22,23,24]. Furthermore, assemblies were controlled for their quality with QUAST v5.3 and Checkm2 v1.1.0 [25,26].

Genomic-based species identification was carried out through GTDB-Tk v.2.5.2 (db release: 220), while antimicrobial and virulence factor genes were predicted by using ABRicate v1.0.1 with CARD (26)/VFDB as reference databases [27,28,29,30]. For gene detection, a minimum threshold of 80% identity and 80% coverage was applied. Chromosomal point mutations associated with antimicrobial resistance were analysed using the PointFinder v.4.1.1 tool (Centre for Genomic Epidemiology, DTU) [31]. The genomic sequences were screened to identify all mutational markers available in the database, using the software’s default parameters (80% identity and a minimum length of 60%).

Furthermore, the presence of plasmids was investigated by comparing the genomic sequences with the PlasmidFinder database (v. 2.1.6) [32] in order to identify potential plasmid-derived sequences among the detected genes.

In addition to 7-gene MLST, an additional cgMLST analysis was conducted for all sequenced genomes by submitting them to the PubMLST resource (https://pubmlst.org/organisms/campylobacter-jejunicoli, accessed on 29 January 2026), selecting the *Campylobacter jejuni*/*coli* PubMLST v2 typing scheme [33,34]. Then, a phylogenetic tree for cgMLST allele profiles was created by the GrapeTree PubMLST plug-in with “MSTreeV2” as the minimum spanning tree generation algorithm [35,36].

## 3. Results

### 3.1. Prevalence of Campylobacter spp. Isolated from Samples

A total of 51 (92.72%) out of 55 samples tested positive for *Campylobacter* spp. In detail, the positivity for *Campylobacter* spp. was distributed as follows: 80% in the neck skin samples and 100% in the cecum and water samples. Moreover, 13 (81.25%) neck skin samples were found exceeding the regulatory limit of 1000 CFU/g.

Out of the 51 bacterial sequenced genomes, ANI-based genomic identification was allowed to assign 33 (64.71%) samples to the species *C. coli* and 18 (35.29%) to the species *C. jejuni*. However, one *C. coli* strain, id: 5988-9, did not satisfy assembly quality check, with a predicted genome contamination of approximately 10%.

*C. jejuni* was detected in 11 (34.37%) out of 32 positive cecum samples and in 7 (43.75%) out of 16 positive neck skin samples. *C. coli* was identified in 21 (65.62%) out of 32 positive cecum samples, in 9 (56.25%) out of 16 positive neck skin samples and in 100% of the 3 positive water samples (Table 1).

Regarding the 32 *C. coli* strains, 10 different MLST sequence types (ST) were detected. Five STs were identified among the *C. jejuni* strains (Table 2).

**Figure 1:**
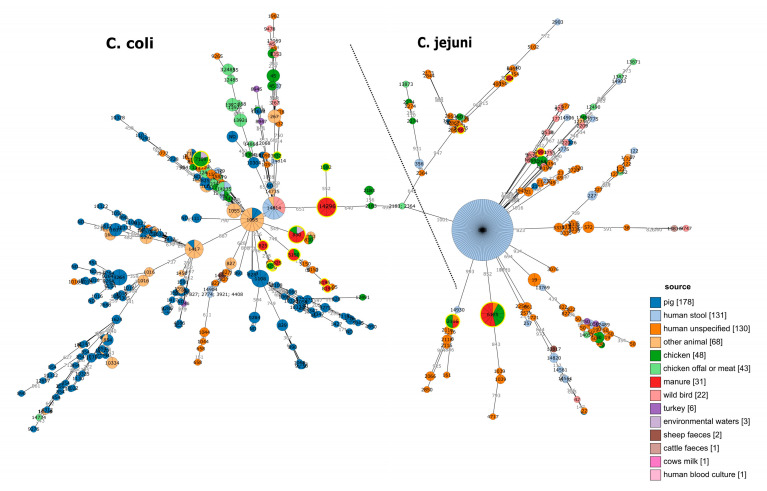
Minimum Spanning Tree for *C. coli/jejuni* cgMLST allele profiles derived from 51 sequenced isolates. Grey numbers: allele differences; node colour: MLST sequence types; node size: sample clusters (cgMLST allele differences ≤ 4); node numbers: sequence types; black dashed line separates *C. coli* from *C. jejuni* strains; red dashed circle: largest cluster among investigated strains.

MLST and cgMLST analyses revealed a highly variable distribution for both collection sites, as shown in Figure 1.

### 3.2. Phenotypic and Genotypic Characteristics of Antimicrobial Resistance

*Campylobacter* spp. isolates showed the following resistance percentages: CHL (2/51) (3.92%), ERY (3/51) (5.88%), GEN (2/51) (3.92%), CIP (44/51) (86.27%), TET (31/51) (60.78%), and ETP (23/51) (45.09%).

The two *Campylobacter* species had antimicrobial resistances that have been reported as follows: *C. jejuni* (18/51) CHL (11.11%), ERY (11.11%), GEN (5.55%), CIP (100%), TET (83.33%), and ETP (38.88%); *C. coli* (33/51) CHL (0%), ERY (3.03%), GEN (3.03%), CIP (72.72%), TET (48.48%), and ETP (48.48%).

The antimicrobial-resistance test results for 51 *Campylobacter* spp. isolates and the related positive samples are presented in Table 1. Out of 51 strains, 18 (35.29%) isolates showed MDR profiles.

Regarding the genomic analysis, antimicrobial resistance gene prediction shows a limited number of predicted resistance genes and a more resistant profile for *C. jejuni* strains (average of 5.8 genes) compared to *C. coli* (average of 3.3 genes).

Overall, 60.78% of the strains analysed (31/51) had the *tet(O)* gene involved in tetracycline resistance. The *cmeA* gene was predicted for 45.09% of the strains (23/51), *cmeB* and *cmeC* for 98.03% (same 50/51 strains), while *cmeR* was predicted for all 18 *C. jejuni* strains and for none of the *C. coli* strains. These four genes encode the subunits of the CmeABC efflux pump, which confers multidrug resistance, and its regulator.

To further investigate the absence of specific constitutive genes, a secondary analysis was carried out by lowering the detection thresholds to 60% identity and 60% coverage. This new analysis enabled the identification of gene variants that had been excluded by the more stringent 80% threshold used in the primary analysis (Supplementary Table ST9). In detail, using these less restrictive parameters, the *cmeA* and *cmeR* genes were detected in all isolates with a coverage of over 76%.

In silico analysis of chromosomal point mutations revealed a strong correlation between phenotypic resistance to ciprofloxacin and the presence of the T86I mutation in the *gyrA* gene. All phenotypically resistant isolates showed this specific substitution, with the exception of one *C. coli* strain (8184-2). In contrast, 9 out of 10 strains that were phenotypically sensitive to ciprofloxacin lacked the T86I mutation.

Another interesting finding from the study of chromosomal point mutations concerns resistance to macrolides. In particular, genomic analysis revealed the presence of the A2075G transition in the 23S rRNA gene in a *C. coli* isolate (5987-3). The isolate exhibited phenotypic resistance to erythromycin, while remaining susceptible to clarithromycin. Furthermore, four isolates exhibiting phenotypic resistance to either erythromycin or clarithromycin lacked known 23S rRNA mutations; however, all four harboured the complete CmeABC operon and the *cmeR* regulator.

The *OXA-450* gene, an *OXA-61-like beta-lactamase* gene, was detected in 78.43% of analysed strains and the *OXA-466* gene, an *OXA-184-like beta-lactamase* gene, was predicted for only two (3.92%) *C. jejuni* strains.

### 3.3. Genotypic Virulence Characteristics and Plasmid Detection Analysis

The screening of the isolates obtained from the Virulence Factor Database (VFDB) revealed a complex set of virulence factors associated with motility, adhesion, and toxin production. The primary flagellin genes (*flaA* and *flaB*) were detected in only one (1.96%) isolate (strain 6163-1). However, a secondary analysis using lower thresholds (60% identity and coverage) revealed the presence of the *flaA* gene in 16 additional isolates (Table ST10), with coverage greater than 61%. In contrast, the *flgB*, *flgG*, *fliM*, and *fliS* genes, which encode the basal body and the structural components of the flagellar apparatus, were identified in all strains (100%; 51/51). The complete Cytolethal Distending Toxin (CDT) gene cluster, which involves the *cdtA*, *cdtB*, and *cdtC* genes, was present in 35.29% (18/51) of the isolates. Specifically, all *C. jejuni* strains harboured the complete cluster, whereas *C. coli* isolates lacked any catalytic subunits.

The fibronectin-binding protein gene (*cadF*), related to universal adhesin, was identified in 100% (51/51) of the strains. In contrast, the surface lipoprotein (*jlpA*) showed a much lower prevalence, being detected in 35.29% (18/51) of the isolates, and all *C. jejuni* strains. Regarding invasiveness, the *Campylobacter* invasion antigen B gene (*ciaB*) was highly conserved, being detected in 98.04% (50/51) of the isolates, with strain 5988-9 representing the sole exception.

Plasmid detection analysis through the PlasmidFinder tool did not reveal any plasmid-related sequences.

**Figure 2:**
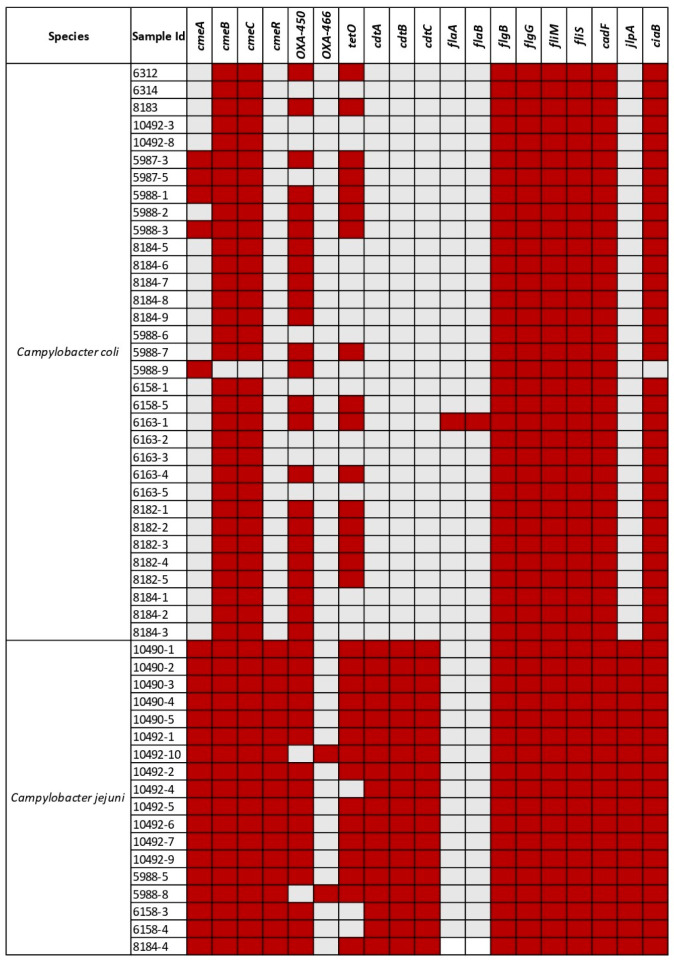
Distribution of antimicrobial resistance and virulence genes in *C. coli* and *C. jejuni* strains (data obtained assuming 80% coverage and 80% identity). Red is for “detected” and white is for “not detected”.

The detected antimicrobial resistance and virulence genes are reported in Figure 2.

## 4. Discussion

This study shows a high prevalence of *Campylobacter* spp. across the sampled matrices, with 92.72% of samples testing positive, confirming the widespread circulation of this pathogen on poultry carcasses and within the poultry slaughterhouse environment [37]. Notably, 13 (81.25%) out of 16 neck skin samples exceeded the regulatory limit of 1000 CFU/g. These high positivity rates and non-compliant microbial loads suggest that even small-scale poultry slaughterhouses may be a significant source of contamination, potentially facilitating the spread of *Campylobacter* throughout the food chain.

In this context, the EFSA and ECDC report on the *Campylobacter* process hygiene criterion highlights similarly concerning findings, indicating that the number of samples exceeding the limit was significantly high both in official samples (24.3%) and in food business operator samples (16.4%) in 2024 [8].

In our study, *C. coli* was the predominant species across all sample types, and its predominance over *C. jejuni* is unusual compared to previous reports in the scientific literature [16]. Our findings could be attributed to direct or indirect contact with pigs, which are a known reservoir of *C. coli* [38].

This hypothesis is further supported by the distribution of STs identified in our work. In particular, ST830 and ST7159, detected in our study, have previously been reported among *C. coli* isolates recovered from swine, highlighting the role of pigs as an important reservoir for these lineages [39].

Interestingly, strains 10490-1, 10490-2, 10490-3, 10490-4, 10490-5, 10492-1, 10492-2, 10492-5, 10492-6, 10492-7, 10492-9 (site B), and 5988-5 (site A) clustered together with a previously sequenced strain from a human faecal sample (PubMLST Id: 151657). The remaining STs identified in this study are commonly associated with poultry [3,17].

Regarding phenotypic antimicrobial resistance, all *C. jejuni* isolates (100%) and 72.72% of *C. coli* isolates were resistant to fluoroquinolones. These data confirm the well-established resistance to this class of antibiotics, which is of particular concern and has led the World Health Organization (WHO) to include fluoroquinolone-resistant *Campylobacter* in the list of high-priority antibiotic-resistant pathogens for the research and development of new antimicrobial agents [12].

Tetracycline resistance was observed in 83.33% of *C. jejuni* isolates and 48.48% of *C. coli* isolates, with percentages consistent with those reported in the literature. In contrast to previous reports, macrolide resistance was detected in only 3 (5.88%) out of 51 isolates [16]. It is interesting to note that a recent study examining antimicrobial resistance in human clinical isolates of *C. jejuni* and *C. coli* from Croatia reported a relatively lower level of resistance. In fact, the authors reported ciprofloxacin resistance of 43% and 7%, and tetracycline resistance of 45% and 26.7% for *C. jejuni* and *C. coli*, respectively [40]. Apart from the different number of isolates tested in these studies, the remarkable level of resistance of poultry isolates could be explained by the inappropriate use of antimicrobials during breeding.

Carbapenem resistance was observed in 45.09% of the isolates (23/51). It is alarming, as carbapenems are classified as critically important antibiotics and are recommended for treating invasive *Campylobacter* infections in humans, particularly in cases of complications or the failure of first-line therapies. These results may suggest the acquisition of resistance mechanisms by *Campylobacter* as a consequence of prolonged selective pressure [12]. Resistances observed in our study agree with those described in the EFSA-ECDC 2025 published report [41]. Only 2 (3.92%) out of 51 isolates, both identified as *C. jejuni*, were resistant to chloramphenicol.

Overall, 35.29% of the isolates (18/51) exhibited a multidrug-resistant (MDR) profile, being resistant to three or more antimicrobial classes. Although findings were lower than those reported in some previous studies, the observed prevalence of MDR *Campylobacter* isolates still represents a serious public health issue [12].

Genomic analysis confirmed that 60.78% of the isolated strains (31/51) harboured the *tet(O)* gene, which represents a primary mechanism of tetracycline resistance in *Campylobacter* [42]. This gene encodes a ribosomal protection protein that recognises a site on the bacterial ribosome, binds to it, and induces a conformational change, leading to the release of the bound tetracycline molecule [43]. According to a recent review, this gene is often carried by plasmids [44].

The CmeABC efflux pump allows *Campylobacter* spp. strains to be resistant to a broad spectrum of antimicrobial agents and is also essential for the colonisation of the animal intestinal tract through the mediation of bile resistance. This is one of the main environmental adaptation systems of the *Campylobacter* genus. The *CmeA* gene encodes for a periplasmic fusion protein, the *cmeB* gene for the inner membrane transporter, and the *cmeC* gene is an outer membrane protein. The CmeABC overexpression results in increased resistance to ciprofloxacin, norfloxacin, cefotaxime, fusidic acid, and erythromycin [45]. Only four (12.12%) of the 33 strains of *C. coli* have genes encoding all three subunits, suggesting that they have a fully functional efflux pump, while the remaining 29 have only the *cmeB* and *cmeC* genes, except for one strain that has only the *cmeA* gene. Interestingly, all *C. jejuni* strains harbour the three subunit genes. Moreover, all these strains also carry the *cmeR* gene, a transcriptional repressor that modulates pump activity and is specific to this *Campylobacter* species [46].

The detection of the *cmeA* and *cmeR* genes, obtained by lowering the identity and coverage thresholds, suggests the presence of non-canonical allelic variants. Our results demonstrate that the structural model of CmeABC is conserved throughout the collection, but its polymorphism requires less stringent bioinformatic filters to avoid false negatives.

Furthermore, our genomic analysis of chromosomal mutations confirms the phenotypic results. The high level of resistance to ciprofloxacin observed in the isolates appears to be primarily due to the T86I substitution in the *gyrA* gene, while resistance to erythromycin seems to be partially associated with the A2075G transition in the 23S rRNA gene, suggesting that other mechanisms also contribute to the observed phenotype.

The presence of the complete CmeABC operon, in synergy with mutations in the *gyrA* or 23S genes, enables the isolate to achieve a high resistance level [45,47]. The presence of a phenotypically resistant isolate lacking mutations in the *gyrA* or 23S genes suggests that, in some cases, the presence of the CmeABC pump may be sufficient to confer phenotypic resistance even in the absence of chromosomal substitutions [48].

Overall, this multi-level bioinformatic approach provides a more accurate correlation between the efflux system genotype and the observed resistance phenotype.

With regard to beta-lactam resistance, we found the presence of the *blaOXA-450* gene in 78.43% (40/51) of strains, in agreement with previous reports in the literature for strains isolated from poultry in Italy [49].

Only two strains of *C. jejuni* (3.92% of the total) were predicted to harbour the presence of the *blaOXA-466* gene, which has been reported sporadically in the literature for *Campylobacter* spp. strains isolated from birds, and appears to be less common than the previously mentioned strain [50].

Based on the plasmid detection analyses performed, it was not possible to establish with certainty whether the detected resistance and virulence genes were located on plasmids, other mobile genetic elements, or the bacterial chromosome. Although in silico screening did not identify any known plasmid sequences, the exact genomic localization of these markers was not further defined.

Genomic analysis of virulence factors reveals a heterogeneous profile among the 51 isolates. While the initial VFDB screening suggested a near-total absence of flagellin genes (*flaA, flaB*), detected via VFDB, the universal conservation of flagellar structural components suggests that motility- and secretion-related functions could potentially be preserved. In such cases, it may be necessary to evaluate and adjust the screening parameters, as a standard level of specificity could overlook significant genetic variations, for instance, applying lower identity and sequence coverage thresholds (60%), the *flaA* gene was identified in 17 additional isolates, with sequence coverage levels greater than 61%. This may indicate that the isolates harbour divergent flagellins not detected by the current bioinformatic screening while retaining the structural foundations necessary for motility and secretion. However, further phenotypic testing would be required to confirm the functionality of these systems. The widespread presence of the *ciaB* gene further supports a conserved invasive potential across all isolates.

With regard to interaction with the host cell, the detection of the *cadF* gene in all strains confirms its role as the primary adhesin in both *C. jejuni* and *C. coli*. In contrast, *jlpA* gene was detected at low frequency (35.29%), suggesting a minor and strain-dependent role in adhesion. Toxigenic potential varied significantly among the isolates: the complete cdtABC operon was identified in only 35.29% of the isolates and was restricted to *C. jejuni*, whereas all *C. coli* strains lacked the catalytic subunits, suggesting attenuated toxin-mediated pathogenicity.

Our findings suggest distinct virulence strategies between the two species. *C. coli* pathogenicity appears to rely predominantly on adhesion and invasion mechanisms, with limited contribution from cytotoxicity, whereas *C. jejuni* combines invasive capacity with CDT-mediated damage.

## 5. Conclusions

In conclusion, the high prevalence of *Campylobacter* spp., coupled with the widespread occurrence of antimicrobial resistance, and the detection of key resistance determinants underscores the potential consumer risks associated with poultry meat.

Moreover, the presence of pathogenicity-related genes highlights the potential of these strains to cause human disease, thereby increasing the associated public health concerns. Our study confirms the widespread circulation of various STs that may be common to poultry, pigs, and humans, and highlights the urgent need for stringent hygiene practices in primary production and at slaughterhouses. In addition, continuous monitoring and targeted interventions along the poultry production chain are required to limit the spread of pathogenic and antimicrobial-resistant *Campylobacter* strains and to safeguard public health. Finally, consumer awareness of the risk of infection from handling raw chicken or eating undercooked meat should be greatly enhanced through public campaigns or simple warnings on meat packaging.

## Figures and Tables

**Table 1:** Number and type of samples resulted positive to *C. coli* and *C. jejuni* and related phenotypic resistances to single antimicrobial agents.

Samples					Antimicrobial Resistance					
Samples	n. of Samples	n. of Positive Samples (%)	Species (n. of Positive)		CHL	ERY	GEN	CIP	TET	ETP
Neck skin	20	16 (80%)	*C. jejuni*	(7/16)	1/7 (14.28%)	0%	0%	7/7 (100%)	5/7 (71.43%)	4/7 (57.14%)
			*C. coli*	(9/16)	0%	1/9 (11.11%)	0%	9/9 (100%)	8/9 (88.88%)	2/9 (22.22%)
Cecum	32	32 (100%)	*C. jejuni*	(11/32)	1/11 (9.09%)	2/11 (18.18%)	1/11 (9.09%)	11/11 (100%)	10/11 (90.9%)	3/11 (27.27%)
			*C. coli*	(21/32)	0%	0%	1/21 (4.76%)	12/21 (57.14%)	6/21 (28.57%)	13/21 (61.9%)
Water	3	3 (100%)	*C. jejuni*	(0/3)	-	-	-	-	-	-
			*C. coli*	(3/3)	0%	0%	0%	3/3 (100%)	2/3 (66.66%)	1/3 (33.33%)

**Table 2:** *C. coli* and *C. jejuni* strains isolated from samples collected at two slaughterhouses located in Picerno (site A) and Abriola (site B) and related phenotypic resistances to single antimicrobial agents.

Samples					Antimicrobial Resistance					
ID Strain	Sample	Species	MLST ST	Site	CHL	ERY	GEN	CIP	TET	ETP
5988-1	Cecum	*C. coli*	1595	A	S	S	S	R	R	R
5988-2	Cecum	*C. coli*	8195	A	S	S	S	R	R	R
5988-3	Cecum	*C. coli*	825	A	S	S	S	R	R	R
5988-5	Cecum	*C. jejuni*	6175	A	S	S	S	R	R	S
5988-6	Cecum	*C. coli*	830	A	S	S	S	R	S	S
5988-7	Cecum	*C. coli*	8195	A	S	S	S	R	R	R
5988-8	Cecum	*C. jejuni*	7991	A	S	S	S	R	R	R
5988-9	Cecum	*C. coli*	-	A	S	S	S	R	S	S
6163-1	Cecum	*C. coli*	5150	B	S	S	S	R	R	R
6163-2	Cecum	*C. coli*	830	B	S	S	R	R	S	S
6163-3	Cecum	*C. coli*	830	B	S	S	S	R	S	S
6163-4	Cecum	*C. coli*	5150	B	S	S	S	R	R	R
6163-5	Cecum	*C. coli*	830	B	S	S	S	R	S	S
6158-1	Neck skin	*C. coli*	830	B	S	S	S	R	S	S
6158-3	Neck skin	*C. jejuni*	2116	B	S	S	S	R	S	R
6158-4	Neck skin	*C. jejuni*	2116	B	S	S	S	R	S	R
6158-5	Neck skin	*C. coli*	5150	B	S	S	S	R	R	R
5987-3	Neck skin	*C. coli*	832	A	S	R	S	R	R	R
5987-5	Neck skin	*C. coli*	825	A	S	S	S	R	R	S
6312	Water	*C. coli*	860	A	S	S	S	R	R	R
6314	Water	*C. coli*	830	A	S	S	S	R	S	S
8182-1	Neck skin	*C. coli*	7159	A	S	S	S	R	R	S
8182-2	Neck skin	*C. coli*	7159	A	S	S	S	R	R	S
8182-3	Neck skin	*C. coli*	7159	A	S	S	S	R	R	S
8182-4	Neck skin	*C. coli*	7159	A	S	S	S	R	R	S
8182-5	Neck skin	*C. coli*	1582	A	S	S	S	R	R	S
8183	Water	*C. coli*	7159	A	S	S	S	R	R	S
8184-1	Cecum	*C. coli*	14296	A	S	S	S	S	S	R
8184-2	Cecum	*C. coli*	14296	A	S	S	S	R	S	S
8184-3	Cecum	*C. coli*	14296	A	S	S	S	S	S	R
8184-4	Cecum	*C. jejuni*	354	A	S	S	S	R	R	R
8184-5	Cecum	*C. coli*	14296	A	S	S	S	S	S	R
8184-6	Cecum	*C. coli*	14296	A	S	S	S	S	S	R
8184-7	Cecum	*C. coli*	14296	A	S	S	S	S	S	R
8184-8	Cecum	*C. coli*	14296	A	S	S	S	S	S	R
8184-9	Cecum	*C. coli*	14296	A	S	S	S	S	S	R
10490-1	Neck skin	*C. jejuni*	6175	B	S	S	S	R	R	R
10490-2	Neck skin	*C. jejuni*	6175	B	S	S	S	R	R	R
10490-3	Neck skin	*C. jejuni*	6175	B	R	S	S	R	R	S
10490-4	Neck skin	*C. jejuni*	6175	B	S	S	S	R	R	S
10490-5	Neck skin	*C. jejuni*	6175	B	S	S	S	R	R	S
10492-1	Cecum	*C. jejuni*	6175	B	S	S	S	R	R	S
10492-2	Cecum	*C. jejuni*	6175	B	S	S	S	R	R	S
10492-3	Cecum	*C. coli*	825	B	S	S	S	S	S	S
10492-4	Cecum	*C. jejuni*	2116	B	R	S	S	R	S	R
10492-5	Cecum	*C. jejuni*	6175	B	S	S	S	R	R	S
10492-6	Cecum	*C. jejuni*	6175	B	S	R	S	R	R	S
10492-7	Cecum	*C. jejuni*	6175	B	S	S	R	R	R	S
10492-8	Cecum	*C. coli*	825	B	S	S	S	S	S	S
10492-9	Cecum	*C. jejuni*	6175	B	S	R	S	R	R	S
10492-10	Cecum	*C. jejuni*	2863	B	S	S	S	R	R	S

## Data Availability

The original contributions presented in this study are included in the article. Further inquiries can be directed to the corresponding authors.

## Supplementary Materials

The following supporting information can be downloaded at: https://www.mdpi.com/article/10.3390/pathogens15060585/s1, Table ST1: Samples metadata and phenotypic antimicrobial susceptibility; Table ST2: Taxonomic classification of the isolates; Table ST3: Genome assembly quality assessment and general statistics; Table ST4: MLST profiles; Table ST5: Detailed assembly metrics and contig statistics; Table ST6: Acquired Antimicrobial Resistance (AMR) genes identified via ABRicate (80% identity and coverage thresholds); Table ST7: Virulence factors identified via VFDB screening (80% identity and coverage thresholds); Table ST8: Chromosomal point mutations identified via PointFinder. Table ST9: Acquired Antimicrobial Resistance (AMR) genes identified via ABRicate using reduced thresholds (60% identity and coverage); Table ST10: Virulence factors identified via VFDB screening using reduced threshold (60% identity and coverage).
